# A Layered Approach for Robust Spatial Virtual Human Pose Reconstruction Using a Still Image

**DOI:** 10.3390/s16020263

**Published:** 2016-02-20

**Authors:** Chengyu Guo, Songsong Ruan, Xiaohui Liang, Qinping Zhao

**Affiliations:** State Key Lab of Virtual Reality Technology and Systems, Beihang university, Xueyuan Road No.37, Haidian District, Beijing 100000, China; guochengyu@buaa.edu.cn (C.G.); ruan.answer@gmail.com (S.R.); zhaoqp@vrlab.buaa.edu.cn (Q.Z.)

**Keywords:** body part detection, pose estimation, spatial pose reconstruction, deep model

## Abstract

Pedestrian detection and human pose estimation are instructive for reconstructing a three-dimensional scenario and for robot navigation, particularly when large amounts of vision data are captured using various data-recording techniques. Using an unrestricted capture scheme, which produces occlusions or breezing, the information describing each part of a human body and the relationship between each part or even different pedestrians must be present in a still image. Using this framework, a multi-layered, spatial, virtual, human pose reconstruction framework is presented in this study to recover any deficient information in planar images. In this framework, a hierarchical parts-based deep model is used to detect body parts by using the available restricted information in a still image and is then combined with spatial Markov random fields to re-estimate the accurate joint positions in the deep network. Then, the planar estimation results are mapped onto a virtual three-dimensional space using multiple constraints to recover any deficient spatial information. The proposed approach can be viewed as a general pre-processing method to guide the generation of continuous, three-dimensional motion data. The experiment results of this study are used to describe the effectiveness and usability of the proposed approach.

## 1. Introduction

In recent years, because powerful resources have spread through the Internet with different dimensions, how to capture key information from a large amount of data is of interest to researchers. Vision data, which are typically still images or video clips, are a primary form of data that are used to record scene or human activity information. Pedestrian detection and pose reconstruction are typically used to capture the key information concerning pedestrians or sportsmen due to their practical applications in scene surveillance, motion animation reconstruction and intelligent robot simulation or navigation. The problem with this process can always be presented as follows: with an unrestricted vision input, which is typically sparse, deficient and multi-scale, the three-dimensional joint information of a real human is difficult to recover precisely using finite resources.

To address this problem, several key modules of the general detection methods must be reviewed. As an indispensable component, feature selection can be used to determine the most discriminative information of a human body in an image. Common features, such as a contour or edge histogram descriptor in [1,2], a local intensity feature descriptor, such as SIFT (scale-invariant feature transform) in [3], HOG (histogram of oriented gradients) in [4,5,6,7] and other feature descriptors, such as a color- or texture-based descriptor, can be used to distinguish the difference between each body part and the background in an image while simultaneously maintaining a tolerably-internal variant of each component. The classifier selection directly determines the detection results. General classifiers, which are typically divided into discriminative classifiers and generative classifiers in [8,9,10,11,12], classify the regions of an image using a given window. In addition to these two basic pipelines of detection, additional methods are typically used to address the constraints of the human body and to capture both the deformation of each body part and the relationship between each body part. Although the general framework can complete the detection successfully, the task of reconstructing virtual, three-dimensional information in detail for each joint remains an important issue. The difficulty of this process is caused by three critical and required steps in the process: (1) selecting and building a model that can detect each body part despite any noise or occlusion; (2) locating joint positions in detail in the vision image domain with any deficiency caused by a previous component; and (3) reconstructing ground-truth, three-dimensional pose information using low-dimension, untrustworthy information.

This study attempts to address the problem discussed above and to reconstruct practicable three-dimensional data to guide the generation of virtual human animation and control intelligent machines or robots in real applications. The proposed framework is based on the three-layer process described above, which can efficiently distinguish a visible body part, determine any occlusion and estimate and recover three-dimensional joint positions precisely. The recovered joint information will be mapped by a perspective model using image coordinates, and a multi-constrained, strong, fault-tolerant, iteration process is used to recover joint information, which includes both visible and invisible parts of the body.

As discussed above, the contribution of this article can be summarized as follows. A robust hierarchical deformable model with Markov random fields is used to efficiently incorporate a deep model based on convolutional neural networks (CNN) to recover planar joint position information by considering complex hidden relations and recovering fuzzy and deficient information. The proposed deep model can accurately refine each body joint position using regional results. A framework to reconstruct a three-dimensional human pose is presented by integrating the proposed deep model into a multi-constrained, strong, fault-tolerant, perspective model, which can effectively recover any ambiguities. The results of the proposed framework can directly guide the generation of virtual motion data and apply to other applications.

## 2. Related Work

As vision detection and tracking technologies develop, more artificial intelligence methods are being used in the contexts of pedestrian detection, pose estimation and motion reconstruction. Typical methods, including appearance-based, spatial, temporal, behavior-based and deep network models, can be adapted for use in human pose estimation as shown in [13,14]. Appearance-based models typically use image data to acquire the *a priori* knowledge of the human body in a specific scene, which typically requires two steps: feature extraction and classification. As discussed above regarding general feature descriptors, the intensity of a still image is the most common feature that is used to distinguish a part of the human body from the background or other body parts in an image; such methods include HOG and SIFT. The local detail of an image can also be described using other features, such as color and texture in [15]. However, optical flow in [16] could produce a better approximation of continuous motion. The features are then codified by classifiers, such as SVM, example-based methods and salient-point analysis, to parse the information describing the position of the human body in the images. Although many methods are now available, the appearance-based model remains common when extracting key image information.

In recent years, combining two or more methods to mitigate background noise and address the relationship between each body part has become more popular. Because they are representative of the spatial model, pictorial structures are typically adapted to model the dependence of each body part, which encodes the configuration of the human body in a soft way. Each part of the structure remains detected by the appearance-based model. The work in [17] proposed a Bayesian framework that integrates the pictorial structure model using latent variables and discriminative 2D part detectors based on HOGs. Then, inference was used with evolutionary algorithms to reconstruct the human pose. The work in [18] used two-layered, random forests as joint regressors to obtain good part templates by combining the pictorial structure with the proposed joint regressors to increase the classifier discrimination. The work in [19] introduced a novel, 3D, pictorial, structure model to resolve the ambiguities of mixed body parts of multiple human bodies after triangulation in addition to those created by false-positive body part detections. Except for the spatial model, the temporal and behavior-based models are integrated in specific scenes, such as when multiple, discrete, input images are present. However, the frameworks described above are typically prevented from using tree-based pose models with a simple binary potential that do not depend on the image input as [20] presents.

Deep networks have recently shown outstanding performances for image classification tasks. Compared to the traditional methods above, deep networks can describe more complex models, allowing them to learn powerful object representations without designing features. This ability allows researchers to build a robust, iterative, regression process from the features extracted from an appearance-based model. Deep architectures for pedestrian detection and pose estimation are motivated by part-based models and are extended from the two-layer model (e.g., a pictorial structure), where the human in an image is expressed as a layered composition of image primitives. Both approaches, however, use the neural networks as local or semi-local classifiers, either over superpixels or at each pixel location. In several common deep-learning networks, convolutional networks (ConvNets) show better performances for many vision tasks as [20,21,22,23] show. The work in [24] attempted to find good qualitative interpretations of high-level features that are represented by several deep models and proved the validity of deep models when used in those applications. The work in [22] used the power of ConvNets for object detection, which classified and precisely localized objects, capturing strong geometric information for classification. The work in [20] formulated a pose-estimation task as a deep neural network(DNN) -based regression problem using body joints, which shows that ConvNets can capture the full context of each body joint. The work in [21] focused on solving the limitation of traditional ConvNet architectures, reducing the computational requirements; it also introduced an architecture that includes a position refinement model that is trained to estimate the joint offset location within a small region of the image, increasing the accuracy of the final joint estimation. The work in [23] presented a real-time, continuous, hand-pose recovery method of markerless, complex, articulable objects from a single depth image. In this method, a ConvNet was used for dense feature extraction and was integrated with an inverse kinematics stage for stable real-time pose recovery. The work in [25] presented an architecture with a multi-layer convolutional network and a modified learning technique that learned low-level features and higher level, weak spatial models for human pose estimation, which focused on more various and unconstrained human poses. ConvNets have been extended into higher dimensional spaces, which capture motion information that is encoded in multiple adjacent frames to extract features from spatial and temporal dimensions for tracking and recognition tasks as [26,27,28] show.

For the state of the art methods mentioned above, deep networks present their specified validity for the vision process. However, on the one hand, for complex scenes, the state of the art methods remain in the development phase, especially dealing with complex pose and reticular occlusion relationships. On the other hand, three-dimensional reconstruction from deficient image planar information is also challenging. A framework to directly provide practicable three-dimensional information to generate virtual motion data and guide the following applications is urgent.

## 3. Method

### 3.1. Overview

**Figure 1:**
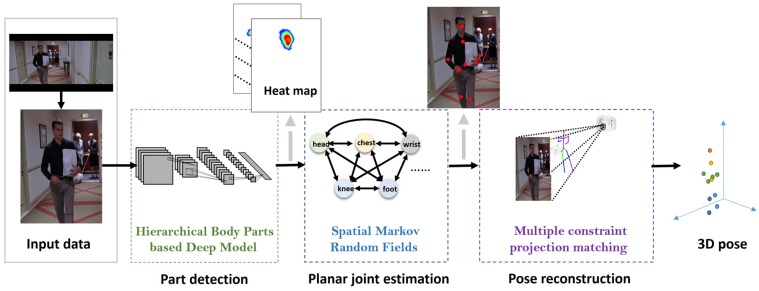
Overview of the framework. With the normalized input, three levels of processing are conducted for part detection, planar pose estimation and spatial pose reconstruction.

To reconstruct a virtual three-dimensional pose, the proposed framework was decomposed into three key layers, as shown in Figure 1. The feature extraction and visualization process ensured that the feature of each body part could be detected and evaluated using a hierarchical body parts-based model and ConvNets, which generate heat maps of the low-level part detection results. Using the rough detection results without invisible parts due to occlusion, a high-level, spatial model of Markov random fields is used to constrain the joint dependency, which provides the global joint distribution. With the model, the joint positions of the human body are precisely estimated, and the invisible parts of the body are managed using prior information. For the final component, planar joint position information is transmitted to a multi-constraint perspective model to map the coordinates of the planar joint position into a virtual three-dimensional space. This framework is detailed below.

### 3.2. Low Level: Hierarchical Body Parts-Based Deep Model

**Figure 2:**
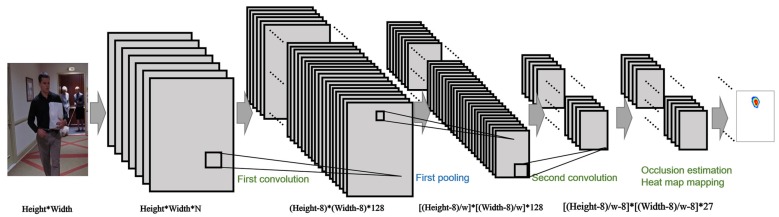
The structure schematic of the deep model at the low level. With two convolution and pooling layers, the scores are mapped to the heat map, which indicates the possible joint distribution of the input image.

At the first stage of the proposed framework, a deep network is used to roughly detect the distribution of visible body parts and approximate the arrangement of invisible parts or complex poses. The structure of the deep model is shown in Figure 2. The input of the architecture is an ordinary RGB image from an experiment database or the Internet, which contains one or more human bodies. The output of this model is a heat map image, which represents the similarity and probability likelihoods of each pixel for each body part. All of the input images are manually corrected to ensure that the size of the human in each image is restricted to be within a certain range. The detection window is also extracted from the entire image to have a size that is marginally larger than the human body in the image.

The image preparation process includes primary feature extraction and visual information maximization. The image data are first translated into the normalized image size and are then transformed from the RGB color space into the YUVcolor space. The three channels of the YVU color space are concatenated into the first channel of the input image data.

In addition to the brightness and color-difference information derived from the original image, certain discriminative information in the image can be identified during the preparation process. The well-known descriptor HOG [4] is typically imported to maintain inter-class variations. However, the general HOG descriptor typically cannot maintain the area well when addressing a part of a body with large variances or rotations. Thus, the gradient is translated into a frequency representation, and features are mapped into an annulus using a Fourier transform in [29]. During this process, the original gradient image is normalized by a smooth convolution kernel function and is then projected to multiple Fourier space. In a different manner, the Fourier basis functions here are composed of different pairs of Fourier space and radial radius scale in our experiments. Six Fourier spaces are chosen, and different radial radius scales with nine pixel gaps are determined. The convolution results by each Fourier basis function are presented as real and imaginary parts for each pixel, which can be organized as the invariant feature descriptors. These invariant features are then formed as Matrix $R_{ijn}$, where *i* and *j* are the height and width of the image input, respectively, and *n* describes the selected Fourier space. All rotation-invariant features are concatenated in the second channel of the input data. Then, the complete feature descriptor we proposed consists of two channels: the characteristic channel and the invariant channel. The characteristic channel is applied to retain the direct appearance information from the brightness or color space in the image, and the invariant channel is used to restore the information from some deformations, such as rotation or warping. After input data pre-processing, information about the human in the image at different resolutions and different rotations is retained for the convolutional layer to extract more detailed variance.

The feature extraction process primarily focuses on detailing the features from the input information. To accomplish this goal, the sliding window-based model [30,31] combined with the hierarchical body part model is proposed to maintain the translation-invariant properties during the feature extraction process. Similar to a general CNN, the net is primarily composed of three types of layers: a convolutional layer, a max-pooling layer and a fully-connected layer. The convolutional and max-pooling layers are based on the standard CNN settings, and the optimized forward propagation approach presented in [30] is based on the full image input. The size of the convolutional filter is 9×9×N, and the output maps are 128. In the max-pooling layer, the output is a set equal to $P_{out}$ of the square maps with a size of w×w. The parameter $P_{out1}$ is described by a function of the convolutional layer’s result $C_{out1}$, where w=4 is the size of the square max-pooling kernel in the proposed experiment.

**Figure 3:**
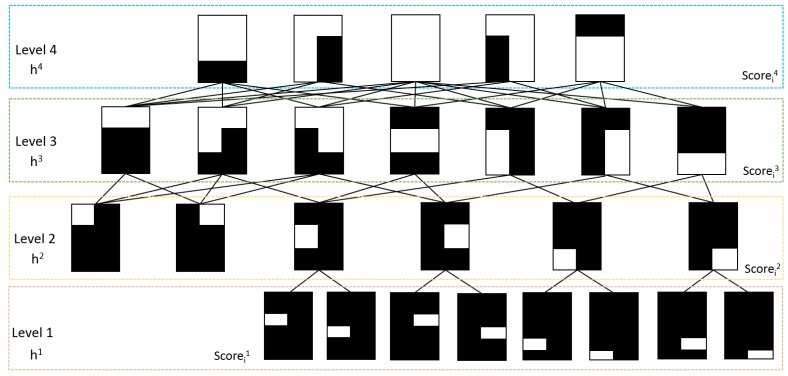
The hierarchical body part templates are used. $h_{i}^{j}$ indicates the visibility state parameters of the *i*-th template in the *j*-th layer. $Score_{i}$ is the detection results from the second pooling layer of the deep model, which indicates the possibility of each visible body part.

For the second layer of the convolutional layer, the detailed information of the different body parts is identified, and the visual state of each human body part should be considered. A hierarchical body part model is used to estimate whether the body parts are occluded. For complex poses and the detailed pose estimation process in the next step, the body part model is used to capture detailed deformation about the joints of the arms and legs compared to the model in [32,33]. Different from general CNN, to identify different body part sizes, a sliding filter size is modified and divided into four hierarchical body part layers with 26 different template filters to address the second convolutional layer of the deep architecture, as shown in Figure 3. The white color in the figure indicates the shape of the filter. In the variant filters with different shapes and sizes, a possible occlusion can be excluded efficiently. For example, with the filter in the shape of the first template at the last row in Figure 3, the local detail of the right upper leg is extracted, and the other body parts are filtered out. Using the 26 filters, the features of different body parts are convoluted using a [(Height-8)/w]×[(Width-8)/w]×27 feature matrix, and a global max pooling is then used with the feature matrix. Then, for each sliding detection window, the scores that are used to measure the similarity between the input and the 26 different levels of body parts can be used to generate the desired heat map.

**Figure 4:**
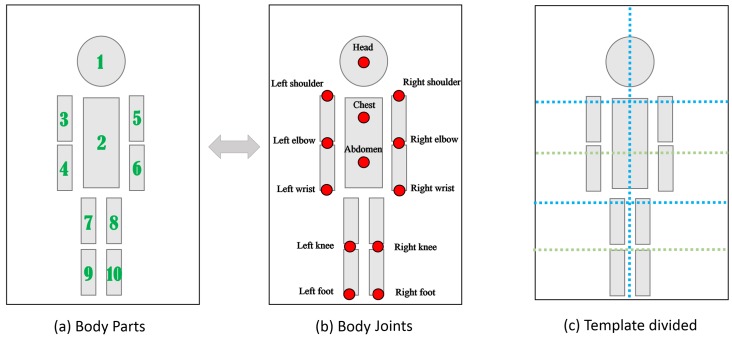
The body structure used in our experiment. (**a**) The fundamental body part structure; (**b**) the joints of the model that need to be estimated and reconstructed in planar and spatial space; (**c**) a division for body parts, which guides the layering of the occlusion templates.

The occlusion estimation and heat map generation process is the final step in generating a rough estimation of a body joint in a planar image. The body part model is shown in Figure 4a, and the skeleton model in the proposed experiments is defined with 13 key joints, as shown in Figure 4b. In this study, we need to build the mapping relationship between the joint of the skeleton models $B_{i}$ and the score map $score_{j}$, where *i* indicates a joint in the skeleton model and *j* indicates the body part in the hierarchical body part model. However, due to occlusion, the score maps of an invisible body part may lead to errors in this process. Therefore, a visibility parameter *H* is required to calculate the weighting of the score maps and to acquire a reasonable joint location distribution heat map.

With the scores of different body part levels $score=\{score_{1},...,score_{N}\}$, the occlusion relations for the body parts must be considered to generate the part detection results. The visibility model in [32] is first redefined to estimate the visibility parameters *H*. The body part model in this process is similar to the body part model shown in Figure 4a and is divided into several templates based on the structure of a typical human body, as shown in Figure 4c. The redefining of the model makes the model more sensitive to arm and leg variations. The visibility parameter *H* is also divided into four levels to serve as a body part template partition relative to the previous visible model: $H=\left\{h^{i}\right\}=\left\{h_{1}^{i},...,h_{L_{i}}^{i}\right\}$, i={1,2,3,4} and $L_{i}=\left\{8,6,7,5\right\}$. The dependencies between each layer are shown in Figure 3. The relationships between the score maps and the visibility parameters are measured by a sigmoid function:

(1) $$\begin{array}{c} h_{j}^{1}=s\left(\lambda_{j}^{1}*score_{j}^{1}+\epsilon_{j}^{1}\right)=\frac{1}{\left(1+e^{-\lambda_{j}^{1}*score_{j}^{1}-\epsilon_{j}^{1}}\right)} \end{array}$$

where *λ* is the weight and *ϵ* is the bias term. For the bottom Layer 1 of the model, the visibility parameters $\left\{h^{1}\right\}$ only depend on the score maps extracted from the deep model. For the higher layers, the influence of the related lower layers must be considered:

(2) $$\begin{array}{c} h_{j}^{i+1}=s\left(\lambda_{j}^{i+1}*score_{j}^{i+1}+\epsilon_{j}^{i+1}+{h^{i}}^{T}*cov_{j}^{i}\right) \end{array}$$

where i=1,2,3,4, $Cov^{i}$ indicates the correlation between two adjacent layers of the visibility parameters and $Cov_{j}^{i}$ indicates the *j*-th column of the matrix. In the training process, the parameters $Cov^{i}$, *λ*, *ϵ* are calculated in the restricted Boltzmann machine training method, as reported in [32,34].

Each joint in the skeleton model is used to create a two-dimensional Gaussian heat map with the mean centered at the possible ground-truth locations. The mean square error is used to minimize the distance between the outputs *H* and Score and the heat map.

### 3.3. High Level: Spatial Markov Random Fields

The high-level layer is structured to re-estimate accurate joint positions using the scored heat map results produced by the low-level layer. In this structure, possible incorrect detections in the past results are also updated, and certain promiscuous body parts are enforced by global pose consistency to present a more reasonable joint distribution. We redefined the spatial models as a fully-connected MRF (Markov random field)-like model to distribute the spatial locations of the thirteen joints defined in Section 3.2. Considering the influence of the body on a given body part $V_{i}$, the locations in relation to other parts $V_{1},...V_{N}$ are considered when determining the distribution of the location of a body part *i*. The graph model node is based on the heat map produced by the deep model; the edges between each pair represent the dependency of the body parts; and the parameters on the edges are correlation parameters that describe the influence between the two body joints. For example, given the location (x,y) of the chest $V_{2}$, the prior location $P{\left(V_{1}|V_{2}\right)}_{\left(x,y\right)}$ indicates the likelihood that the head $V_{1}$ appears at the pixel location (x,y). Then, the likelihood of the head joint distribution can be formalized as follows:

(3) $$\begin{array}{c} P_{v_{i}}\propto P_{v_{i}}^{\lambda }\prod_{v\in V}\left(P_{v_{i}|v}*P_{v}+\epsilon_{v,v_{i}}\right) \end{array}$$

where $P_{i}$ is a parameter of each joint’s unary distribution, *λ* controls the influence of the original unary distribution to the final filtered distribution, $P_{v_{i}|v}$ indicates the conditional prior distribution of the joint $P_{v_{i}}$ when the other joint location *v* is known and $\epsilon_{v,v_{i}}$ is a bias term to ensure that the maxima solution of the function is solvable when previous heat maps are incorrect. The conditional distribution between each of the two joints is determined during training using the datasets shown in Section 4.

The influence of each joint on another joint *i* is different. For example, the relevance of the chest joint and the two elbow joints to the head joint is much higher than the two foot joints; however, relevance between the head and feet does exist to ensure the naturalness and coordination of a given pose. A weight parameter on each edge is necessary to present reasonable relevance between two joints, which are always calculated during the training process. With this modification, the final distribution of a given joint in log space is:

(4) $$\begin{array}{c} \log\left(P_{v_{i}}\right)\propto \lambda \log P_{v_{i}}\underset{v\in V}{\Sigma }\beta_{v,v_{i}}\left(\log P_{v_{i}|v}+\log P_{v}+\log\epsilon_{v,v_{i}}\right) \end{array}$$

The training process uses back-propagation and stochastic gradient descent. Because the spatial model result is not explicitly invariant to scale, the model uses non-maximal suppression to find multiple local maxima from each scale to be the candidate location of the joint, as reported in [25]. With the heat map input, several candidate planar poses of the human body can be efficiently corrected and re-estimated for the subsequent mapping into three-dimensional space.

### 3.4. Spatial Level: Multiple Constraint Projection-Matching-Based Pose Reconstruction

After calculating the planar pose in the image space, the planar pose information can be used to guide the spatial pose reconstruction process. A simple, weak perspective, camera model can be qualified for use when mapping a candidate planar pose onto the ground-truth pose in three-dimensional space. However, there are two key difficulties that must be considered. First, the ambiguity that is generated by deficiencies in the depth information of the planar image produces many mapping relationships when mapping the planar pose onto the spatial pose space. Conversely, because the accuracy of the planar pose estimation must be improved, how to recover incorrect or false positives from the image detection results when reconstructing the spatial pose also must be determined. In this process, a multiple constraint projection-matching method is proposed to recover any deficient dimension information and thus reconstruct the spatial pose to approximate the real ground-truth pose. With an initial spatial pose, we iteratively minimize the projection error to update the spatial pose and camera parameters with multiple constraints based on human engineering and kinematics.

Given favorable initial spatial joint locations, the iterative process will be more efficient and result in a more reasonable achievement. Using a three-dimensional dataset called HumanEva [35] in the proposed experiment, we first cluster typical poses in the dataset and acquire a number of clustering center poses $b_{i}$, which represent essential poses or gestures when combining and generating new gestures. Then, a general spatial pose can be described as:

(5) $$\begin{array}{c} y=\sum_{i=1}^{k}\alpha_{i}\cdot b_{i}+\mu \end{array}$$

where $B=[b_{1},...,b_{i}]$ contain the essential gestures in the dataset of poses. The initial spatial pose is selected from the essential gestures *B*, whose planar projection is most similar to the input planar pose by a set of discrete camera viewpoints.

Considering a group of three-dimensional poses $Y=\{Y_{1},...,Y_{n}\}$, the corresponding planar projection pose *x* can be acquired using a weak perspective projection process:

(6) $$\begin{array}{c} x=\left(I_{P\times P}⊗\left[\begin{array}{cc} s_{x} & 0\\ 0 & s_{y} \end{array}\right]\left[\begin{array}{c} \begin{array}{ccc} 1 & 0 & 0 \end{array}\\ \begin{array}{ccc} 0 & 1 & 0 \end{array} \end{array}\right]R\right)Y+t⊗I \end{array}$$

where $s_{x}$ and $s_{y}$ are the focal distance of the camera in the coordinate directions *x* and *y*, respectively; *t* and *R* are the translation and rotation parameters of the camera, respectively; and ⊗ is the Kronecker product.

To minimize the projection error, a norm function must be introduced to iteratively estimate the spatial pose. However, compared to the L1 norm, the L2 norm is more sensitive to inaccuracies in planar estimation results, because it tends to distribute errors uniformly. Conversely, the L1 norm is robust when recovering information from part deficiencies or incorrect inputs and reduces the influence of these outliers through the iterative process [36]. In this study, the L1-norm error is proposed for minimization as follows:

(7) $$\begin{array}{c} \min_{\alpha }{∥x-(I⊗sR)(\alpha *B+\mu )-t⊗I∥}_{2} \end{array}$$

Different from previous work, to ensure that the acquired gestures correspond to real human structures and kinematic criteria and to reduce the ambiguity created by the projection, multiple constraints are used when solving this minimization problem. The first constraint is the sparse constraint. Inspired by the adjacent properties of low-dimensional gestures in [37,38,39], we enforce sparsity on the parameter *α*, which indicates that the spatial pose can be represented by only a few essential gestures. The sparse constraint can remove incorrect or anthropomorphically-implausible spatial poses and prevent over-fitting. The second constraint is the human-structure constraint, which dictates that the limbs of a human body always satisfy certain length and proportion constraints. These constraints can be formalized as follows: $\left|\right|C_{i}\left(\alpha \cdot B+\mu \right){\left|\right|}^{2}\in \left[Lmin_{i},Lmax_{i}\right]$ and $\left|\right|C_{i}{\left(\alpha \cdot B+\mu \right)||}^{2}/\left|\right|C_{j}\left(\alpha \cdot B+\mu \right){\left|\right|}^{2}\in \left[Pmin_{ij},Pmax_{ij}\right]$, where $C_{i}$ is an operation that calculates the distance between a pair of adjacent joints *i*; Lmin,Lmax are the length constraints; and Pmin,Pmax are proportion constraints. The third constraint is the occlusion constraint. With planar occlusion, described in Section 3.2, the occlusion constraint is constructed to limit the joints that are labeled as invisible to be farther away from the camera than visible joints when they can be projected onto adjacent planar areas. Thus, given certain camera parameters, we can estimate a three-dimensional pose as follows:

(8) $$\begin{array}{cc} & \min_{\alpha }{∥x-(I⊗sR)(\alpha *B+\mu )-t⊗I∥}_{2}-\theta \alpha \\ s.t.\; & Lmin_{i}<||C_{i}\left(\alpha \cdot B+\mu \right){\left|\right|}^{2}<Lmax_{i},i=1,...N\\ & Pmin_{ij}<||C_{i}{\left(\alpha \cdot B+\mu \right)||}^{2}/\left|\right|C_{j}\left(\alpha \cdot B+\mu \right){\left|\right|}^{2}<Pmax_{ij},i=1,...N\\ & \Phi \left(C_{k}\left(\alpha \cdot B+\mu \right)\right)-\Phi \left(C_{l}\left(\alpha \cdot B+\mu \right)\right)>0,for||pr\left(k\right)-pr\left(l\right)||<\delta ,k\in \Omega ,l\notin \Omega \\ & \theta >0 \end{array}$$

where Φ() is a function that calculates the three-dimensional distance from the camera to a given joint location; pr(k) is the projection location of joint *k* in a three-dimensional plane with the given camera parameters; and Ω is the occlusion joint set that is assumed from the output described in Section 3.2.

Similarly, given the three-dimensional pose *Y*, the camera parameters R,t can be acquired as follows:

(9) $$\begin{array}{c} \left\{R^{*},t^{*}\right\}=\arg\min{∥\tilde{x}-\left(I⊗sR\right)\left(\alpha *B+\mu \right)-t⊗I∥}_{2}\; \end{array}$$

The optimization problem alternately updates the 3D pose and the camera parameters. The spatial pose is first initialized, and the initial camera parameters are estimated using the initial pose in Equation (9). With the initial camera parameters, the spatial pose can be re-estimated using Equation (8). This process is repeated until convergence or the maximum number of iterations is reached.

## 4. Results and Discussion

In this section, we evaluate the proposed framework in the following aspects: the detection performance of the hierarchical, body part-based deep model in Section 3.2 for object detection; the pose estimation results in both planar and stereoscopic space in Section 3.3 and Section 3.4. For planar body part detection and pose estimation, Caltech datasets [40], PARSE datasets [41] and INRIA datasets [4] are used to test the body part detection process. Additionally, Frames Labeled In Cinema(FLIC) datasets [42] are used to train the MRF distribution and estimate each body part. Because the scale and input size of two datasets are different, the PARSE and FLIC database have been manually preprocessed to ensure the sizes of the image and human body part are at the same scale and that the label pattern of the these datasets is also unified in the training process. This process guarantees the run-time efficiency of the training process and, at the same time, ensures the consistency of training datasets. In the three-dimensional pose estimation experiment, the CMU database [43] and a few synthetic human poses from the field of motion editing [38,39] are used to extract the essential pose set *B* in Section 3.4. Several types of motion in the HumanEva datasets [35] are selected to evaluate the performance of the proposed methods.

### 4.1. Results on Planar Body Detection and Estimation

In this sub-section, we evaluate the proposed body part detection module using three major datasets: the Caltech dataset, the PARSE dataset and the FLIC dataset. The FLIC dataset is comprised of approximately 5000 images from Hollywood movies with actors in predominantly front-facing, standing poses. The PARSE dataset contains more than 300 full-body, human pose images that are more complex than those in the Caltech and FLIC datasets, because they contain indoor, outdoor and sport scenes with various poses. A total of 1200 negative images in the INRIA dataset are organized for training.

To evaluate the detection performance of the proposed method, the INRIA training dataset is used to train the model, and the Caltech training dataset is used to test the performance of the model. For detailed results on specific image datasets, the miss rates for both the overall training dataset and the divided, unoccluded or partially-occluded pedestrian dataset with 50-pixel-or-taller pedestrians in the Caltech dataset are determined. The miss rates of eight typical methods are chose from the Caltech benchmark results in [40]. The methods are the VJ method in [44], the HOG descriptor in [4], the MFTr + CSS method in [45], the MFTr + Mot method in [45], the DBN-Mut method in [46], the DBN-Isol method in [47], the ChnFtrs method in [48] and the ACF method in [49]. In this experiment, the miss rate is the standard to measure the accuracy of each method. The results of each method with constant false positives per image (FPPI) are shown in Table 1 and exhibit the ability of the method to address the problem of the occlusion of the human body. In the partially-occluded pedestrian dataset, the miss rate of the proposed method is less than or equal to the best currently-available methods, and the performances on the complete and no-occlusion datasets are shown to be considerably superior. In the no occlusion dataset, the miss rate of our method is not outstanding enough relative to the MFTr + CSS, MFTr + Mot, ChnFtrs and ACF methods. Most of the undetected pedestrians by our method in the no occlusion datasets are limited by the size scale of the pedestrian in the image compared to the results in the methods above; some small-scaled pedestrian and background fusion lead to the false cases. In the partial occlusion dataset, compared to the outstanding methods in [45], our method has an evident advantage on relatively normal-scaled pedestrians, but always failed on pedestrians with a small scale. In the heavy occlusion dataset, the overall miss rate is still high. However, most of these methods primarily focus on detecting the entire body of a pedestrian [45], which only broadly consider the body structure; the features and results do not adapt to the proposed framework to further estimate the accurate position of each joints.

**Figure 5:**
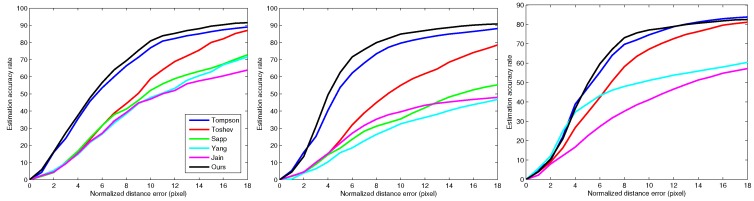
The performance of body joint estimation in planar space on the FLICtest-set. (**a**) Elbow estimation result; (**b**) wrist estimation result; (**c**) foot estimation result.

With the detection results, the proposed method is developed further for planar pose estimation. Figure 5a–c shows the performance of the proposed model using the FLIC test-set on human elbow, wrist and ankle joints, respectively. The model is first trained using the FLIC training datasets. To evaluate the accuracy of the model, we use normalized distance errors (NDEs) to determine whether the joint is correctly estimated. The NDE is a metric that indicates the distance between the estimated location and the ground-truth location in pixels. If the distance is lower than the predefined NDE, the estimated joint location is assumed to be a good result. In this experiment, we compare the proposed method to the best currently-available methods of Tompson [50], Toshev [20], Sapp [42], Yang [6] and Jain [25]. The results show that the proposed method can accurately estimate the joint position in the interval to within six to 12 pixels of the NDE, which is better than the best currently-available methods overall. However, when the NDE is below four, the estimation results must still be improved compared to other methods for certain joints (e.g., knee, foot).

### 4.2. Results on Three-Dimensional Pose Reconstruction

Before building the three-dimensional projection, the model was trained using a given amount of sparse essential poses. In this experiment, the essential poses were identified using the CMU training dataset, which were then used to re-estimate each initial spatial pose to solve the L1-norm, regularized, least square problem. During training, 17 different motion segments were used to train the essential poses and were clustered into 21 cluster centers.

**Figure 6:**
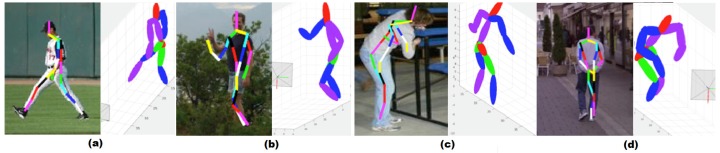
Typical examples for successful and failed three-dimensional pose reconstruction. (**a**,**b**) Two positive results on reconstruction; (**c**,**d**) two typical negative results on reconstruction.

To evaluate the effectiveness of the three-dimensional pose reconstruction, we compared the proposed method against the best currently available methods in a three-dimensional pose estimation area: sequence importance resampling particle filter (SIRPF), Daubney’s method in [51], Simo-Serra’s method in [52] and Wang’s method in [36] on the HumanEva and PARSE datasets, as shown in Table 2. The numbers in each cell are the root mean square error(RMS), and we use units of millimeters as in [52] to evaluate the reconstruction results. The proposed approach produces smaller estimation errors on all joints, particularly with the four leaf joints of the human body. The results show that the proposed approach is adequate for reconstructing planar joint locations. The performance of the framework on boxing data in the HumanEva dataset has also been tested. The planar pose estimation process of the proposed framework is used to directly estimate the planar pose from input images and is then applied to the pose reconstruction. The average planar errors of these two motion types are less than 20, and the final average three-dimensional errors are of the same magnitude as the other two motion types. Figure 6 shows certain successful and failed results of the proposed frameworks in the PARSE dataset. The occlusion arms and legs in Figure 6a,b can be recovered well in most instances. The negative result shown in Figure 6c indicates that major mistakes in planar pose estimation may lead to the failure of the reconstruction process. However, the uncertainties in the body orientation and the initial camera parameters may lead to ambiguous effects on the accuracy of the results, as shown in Figure 6d.

However, we also investigated the influence of each factor on the projection matching process. Table 2 shows the variation of the error rate by changing the influence of the initial spatial pose. We randomly selected 20 initial poses to test the reconstruction error and calculated the average error shown in Table 3. With 20 randomly-selected initial poses in the walking S2 dataset, four reconstruction results are shown to vary widely compared to the standard dataset, which leads to a large average reconstruction error. The results show that the random selection of an initial pose may lead to large errors when a limited number of iterations is used; the errors in the initial pose in the proposed method and the artificially-specified initial pose are approximate, which demonstrates the effectiveness of the initialization process. Table 3 shows the results of the effect of reducing the constraints when solving the L1-norm minimization problem. Without the human structure constraint, the joints’ reconstruction errors accumulate, particularly at the leaf nodes of the human body; the errors of the four limbs are distinctly larger than those of the central nodes, such as the head and chest. Without the occlusion constraint, the results will be relatively large for the given self-occluded inputs. The results show the necessity of each constraint during the projection process.

## 5. Conclusions

This study proposes an efficient framework to reconstruct a spatial human pose using only a still image. In this framework, a two-layer, planar, human detection and pose estimation, deep architecture is proposed to locate each human body part and re-estimate accurate joint locations. In this deep architecture, a hierarchical, body part-based model is used to address the problem of occlusion, and a fully-connected MRF model is used to relocate the detailed positions of the joints. With the planar pose results, a multiple constraint projection matching process is used to map the planar pose information into three-dimensional space. The accuracy and efficiency of addressing various image inputs has been shown experimentally, and the applicability and practicability of the proposed method has been shown in different applications.

In future work, more semantic information regarding the gesture itself should be considered when guiding the location of each joint and generating continuous motion data from several key poses reconstructed using the proposed framework in this study. Using several key photos of a given human motion, the corresponding motion in virtual space can be used to guide the animation or the simulation of a virtual human. Conversely, manual sensor signals should be introduced to simulate the action of a virtual human body from the key poses obtained from visual images.

## Figures and Tables

**Table 1:** The comparison of the miss rates between major excellent or typical methods on the Caltech dataset for the overall data and the three different occlusion level data with false positives per image (FPPI) = 0.1.

Occlusion Level	VJ	HOG	MFTr + CSS	MFTr + Mot	DBN-Mut	DBN-Isol	ChnFtrs	ACF	Our Methods
Overall	99%	90%	81%	78%	82%	84%	81%	81%	80%
No occlusion	96%	72%	58%	51%	58%	62%	60%	60%	57%
Partial occlusion	98%	92%	83%	78%	81%	81%	77%	79%	76%
Heavy occlusion	99%	97%	94%	92%	93%	93%	95%	96%	92%

**Table 2:** The reconstruction of RMS errors on the HumanEva dataset for the two motion types with the other four three-dimensional methods. SIRPF, sequence importance resampling particle filter.

Methods	Walk (S1)	Walk (S2)	Walk (S3)	Jog (S1)	Jog (S2)	Jog (S3)
SIRPF	105.1	105.2	120.7	–	–	–
Daubney [51]	89.3	108.7	113.5	–	–	–
Simo-Serra [52]	99.6	108.3	127.4	109.2	93.1	115.8
Wang [36]	71.9	75.7	85.3	62.6	77.7	54.4
Our framework	68.8	69.9	82.6	59.1	70.4	50.8

**Table 3:** The reconstruction RMS errors on different conditions by changing the initial spatial pose or reducing the constraints.

Condition	Walk (S1)	Walk (S2)	Walk (S3)	Jog (S1)	Jog (S2)	Jog (S3)
Random initial pose	72.1	113.3	105.4	135.5	81.3	81.0
Artificial specified initial pose	70.4	69.2	82.6	58.7	69.1	52.4
Constraints without human engineering	90.1	93.3	102.4	75.5	89.3	76.1
Constraints without occlusion	70.6	70.1	83.0	60.1	75.6	53.2
Our framework	68.8	69.9	82.6	59.1	70.4	50.8
